# Supplementary material for: Assembly of mTORC3 Involves Binding of ETV7 to Two Separate Sequences in the mTOR Kinase Domain
Source: Int J Mol Sci. 2024 Sep 18;25(18):10042. doi: 10.3390/ijms251810042 (PMC11432197; doi:10.3390/ijms251810042)
Supplement: Supplementary file 1 [file ijms-25-10042-s001.zip › ijms-3146508-supplementary.pdf]

Assembly of mTORC3 involves binding of ETV7 to two separate sequences in the mTOR kinase domain.

Jun Zhan, Frank Harwood, Sara Ten Have*, Angus Lamond*, Aaron H. Phillips**, Richard W. Kriwacki**, Priyanka Halder, Gerard C. Grosveld.

Dept. of Genetics, Dept. of Structure Biology, St. Jude Children's Research Hospital

* Center for Gene Regulation and Expression, College of Life Sciences, University of Dundee, Dow Street, Dundee, DD1 5EH, Scotland, UK

**Dept. of Structural Biology, St Jude Children's Research Hospital

Correspondence to Dr. Gerard Grosveld, email: gerard.grosveld@stjude.org

Supplemental Figures

Supplemental Figure 1

A Comparison ETV7-PNT domain of 7 vertebrates: identity 54%, similarity 71%

```
NLLGEGGICKLPGRLRIQPALWSREDVLHWLRWAEQEYSLPCTAEHGFEMNGRALCILTKDDFRHRAPSS--GDVLYELLQYIKTQR Hum
NLLGEGGICKLPGRLRIQPALWSREDVLHWLRWAEQEYSLPCTAEHGFEMNGRALCILTKDDFRHRAPSS--GDVLYELLQYIKTQR Chim
NLLGEGEICKLPGRLRIQPALWSREDVLHWLRWAEQEYSLERTGEHGFEMNGRALCILTKDDFRLRAPGSALGDVLYELLQYIKTQR Dog
QLLGETGICKLPGRLRLQPALWSREDVLHWLRWAEQEYSLPRAPERGFQLNGRALCILTKDDFRRRAPGS--GDVLYELLQFIKTQR Squi
QLIQEEEICKLPGRLRIHPSLWSKEDVIHWLKWAEQEYSLPRTMEQKFEMNGKALCILTKDDFRYRAPSS--GDVLYELLQYIKTQR Tasm
TPADEGHSLALPGRLRIQPSLWSKDDVIHWLRWAEREYSLQQTDESKFEMNGKALCILTKDDFRFRAPGS--GDVLYELLQYIKTQR Chic
SEAVPEELCKLPGRLRINPSLWNKEDVNLWLRWAQREYSLRRADHQGFEMNGKALCLLTKEDFRLRCPSS--GDVLYELLQHVKQQR Zebr
          LPGRLR+ P+LW ++DV  WL WA++EYSL       F++NG+ALC+LTK+DFR R P S  GDVLYELLQ++K QR Overall
```

Comparison ETV7-ETS domain of 7 vertebrates: identity 70%, similarity 84%

```
RLLWDYVYQLLLDTRYEPYIKWEDKDAKIFRVVDPNGLARLWGNHKNRVNMTYEKMSRALRHYYKLNIIKKEPGQKLLFRFL Hum
RLLWDYVYQLLLDTRYEPYIKWEDKDAKIFRVVDPNGLARLWGNHKNRVNMTYEKMSRALRHYYKLNIIKKEPGQKLLFRFL Chim
RLLWDYVYQLLCDTRYEPYIRWEDKNAKIFRVVDPNGLARLWGNHKNRVNMTYEKMSRALRHYYKLNIIKKEPGQKLLFRFL Dog
HLLWDYVYQLLRDARYEPYVRWEDKDAKVFRVVDPNGLARFWGNQKNRVNMTYEKMSRALRHYYKLNIIRKEPGQKLLFRFL Squi
RLLWDYVYHLLSDSKYESYIKWEDKDSKVFRVVDPNGLARLWGNHKNRVNMTYEKMSRALRHYYKLNIIKKEPGQKLLFRFL Tasm
RLLWDYIYQLLSDGRYEPYIKWEDKEAKLFRIVNPHGLAHLWGNHKNRMNMTYEKMSRALRHYYKLNIIKREPGQKLLFRFL Chic
KLLWDYVYQLLSDSRYEAFIRWEDPNDMIFRVVDPNGLARLWGNHKNRANMTYEKMSRALRHYYKLNIIKKETGQRLLFRFL Zebr
 LLWDY+Y LL D +YE +++WED +  +FR+V+PNGLA  WGN KNR NMTYEKMSRALRHYYKLNII++E GQRLLFRFL Overall
```

Comparison of ETV7 sequences between the PNT and ETS domains: identity 5.7%, similarity 19.8%

```
RALVCGPFFGGIFRLKTPTQHSPVPPEEVTGPSQM-------DTRRGHLLQPPDPG--LTSNFGHLDDPGLARWT----------PGKEESLNLCHC-AELGCRTQGVCSFPAMPQ--APIDGRIADC Hum
RALVCGPFFGGAFRLKTPTQHSPVPPEEVTGPSQM-------DTRRGHLLQPPDPG--LTSNFGHLDDPGLARWT----------PGKEESLNLCHC-AELGCRTQGVCSFPAMPQ--APIDGRIADC Chim
QALVCGPFFGGAFRQKMPTQRYPCLLKEGTGPPQL-------APQRDLLQQPSHLG--PASFLSPLS-----KWP----------LGREESLDVFHC-EELGCKTEGDCSSPTMPQ--APIDGRISDC Dog
RALVCRPFLPGTCRQRTRPG-LPQAPEEGIGPFQEA----LLPTAKCLLSQPPALG--LASSFSHLGSP----------------RPARRPLRLSHC-AEPGCRTRGVCSFPRMPR--APIDGRIADC Squi
RALVCGPLFSAPFLRVVPFQQPLCPLEEGNSSLQL-------TSQRVLKTSPWKEG--SSNTSGQLDSISLPRA-----------FFREEPLNLADI----SSGAEVIYSFHRAKQ--DPINGKLADC Tasm
RALMYSPLLNSPFREAKGTGEGLWK--MSWDPPRCSTRFSLSGMDGGAEAAPDAPS--PCLEHTELSPS----------------HSLREPLNLSHPSTEGGCGAESISSFPTAPL--APVDGKIADC Chic
RCAIFSPSSSSGTQISHETTTGPYVPSNGCSTAQM-------QDRTHLFADPEHPQDMQTEIFHHSLKEKMPQISPDKSRQAITVSSTEEPLNLSNK-----------PERPAKNTQPSEATGRIPDC Zebr
++ +  P                       ++ +                 P                                   +++L + +             +           G+++DC Overall
              RCVPVCPVNPNSRAVSA                                          PDKSRQAITV
```

Supplemental Figure 1

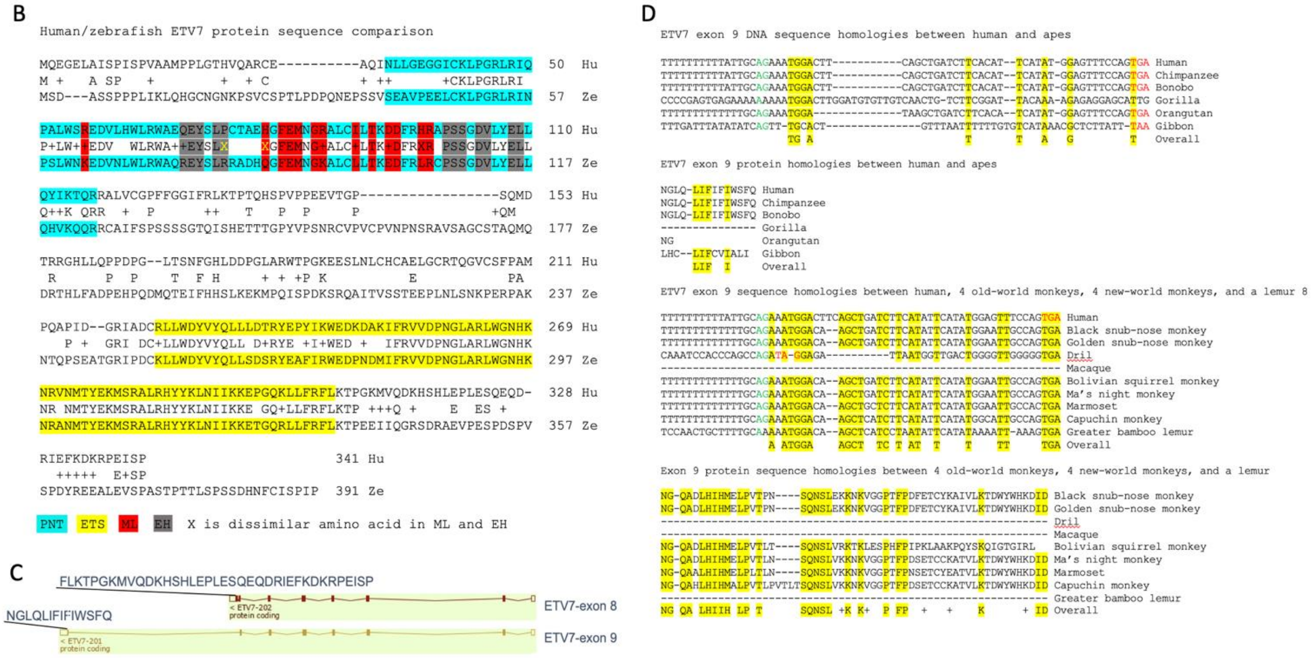

Supplementary Figure S1. Comparison of ETV7 sequences of different vertebrates.

1A. Comparison of ETV7 PNT, ETS, and sequences between the PNT and ETS domains of different vertebrates, including human (Hum), chimpanzee (Chim), dog (Dog), squirrel (Squi), Tasmanian devil (Tasm), chicken (Chick) and zebrafish (Zebr). Highlighted amino acids in yellow indicate identical amino acids in all seven vertebrates.

1B. Comparison of the ETV7 protein sequence of human (Hu) and zebrafish (Ze) ETV7. PNT sequences are highlighted in aquamarine, and ETS sequences are highlighted in yellow. Within the PNT domain, ML sequences are highlighted in red, and EH sequences in grey. X, in yellow, indicates dissimilar amino acids at identical positions in the ML and EH sequences of human and zebrafish.

1C. Genomic structure of ETV7-exon 8 and ETV7-exon 9 showing the protein sequence of the two dissimilar C-termini (https://useast.ensembl.org/).

1D. Comparison of exon 9 DNA and protein sequences of humans and apes and of humans, four old-world, four new-world monkeys, and a lemur. Identical base pairs or amino acids are highlighted in yellow, AG splice acceptor sequences in green, and stop codons in red. + indicates similar amino acids.

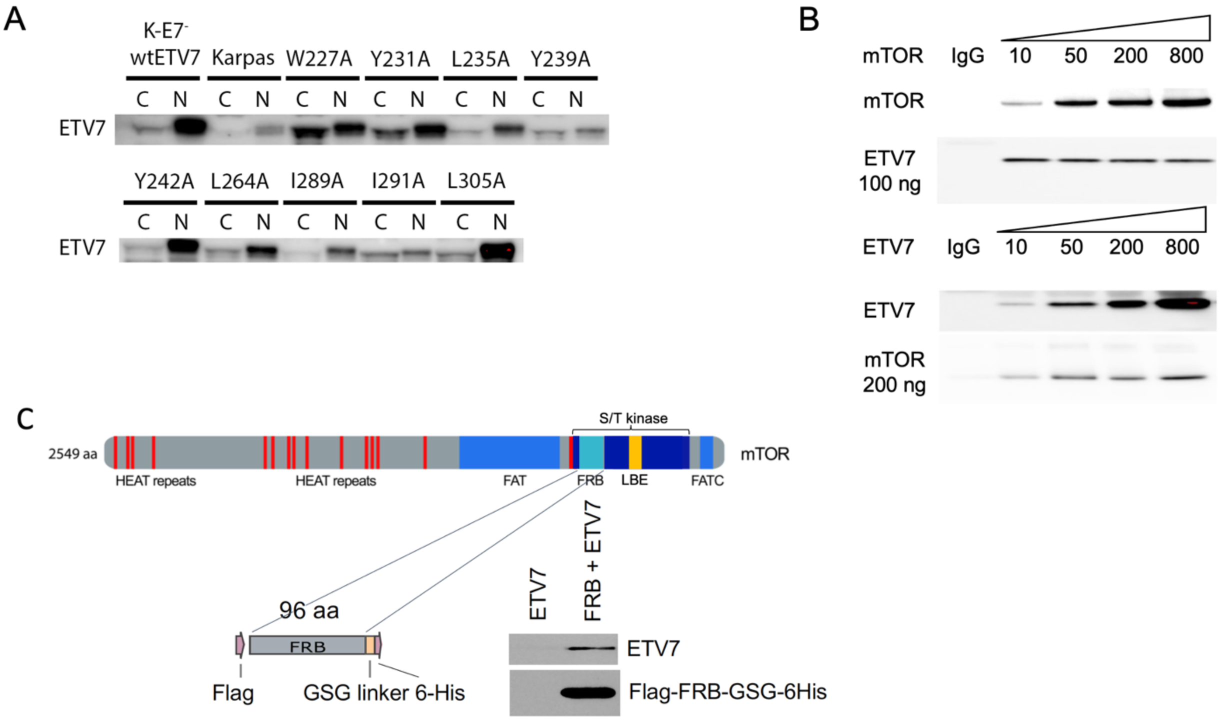

Supplementary Figure S2

Cellular localization of the ETV7 ETS mutants and ETV7-FRB binding *in vitro.*

2A. Western blot of cytoplasmic (C) and nuclear (N) fractions of $KE7^{-}$ cells expressing wtETV7 (wild type), Karpas-299, or $KE7^{-}$ cells expressing ETV7 carrying mutant ETS domains (W227A, Y231A, L235A, Y242A, L264A, I289A, I291A, L305A).

2B. *In vitro* association of purified mTOR and ETV7. Increasing amounts of mTOR were incubated overnight at 4 ° C with a steady amount of ETV7 (100 ng, top panel), and increasing amounts of ETV7 were incubated with a steady amount of mTOR (200 ng, bottom panel). Immunoblots of these ETV7 IPs (top panel) and mTOR IPs were probed for mTOR and ETV7.

2C. The top drawing shows the position of the 96 aa FRB fragment within the mTOR kinase domain (s/t kinase). Below is a blow-up drawing of the Flag-FRB-GSGlinker-6-His fragment, which was used in ETV7 binding experiments. On the right is an immunoblot of purified ETV7 and purified ETV7 + FRB protein, associated overnight at 4 ^{0}C *in vitro*, and pulled down on Ni-NTA beads, probed for Flag and ETV7. Heat repeats are in red, the FAT domain in blue, the FRB domain in Aqua, the LBE sequences in yellow and the

FATC domain in blue. The FLAG sequence is in Pink, the FRB fragment in grey, the GSG linker in yellow and the 6-His sequence in pink.

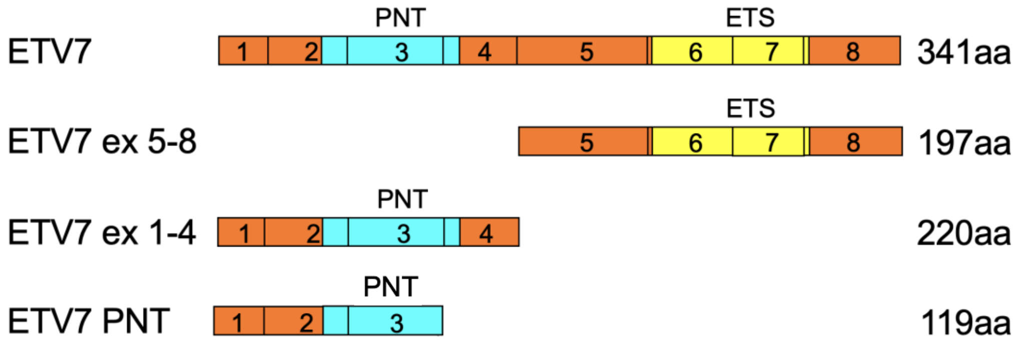

Supplemental Figure S3

Schematic showing different ETV7 deletions used for binding experiments with FRB and LBE protein fragments. The PNT domain is highlighted in aqua and the ETS domain in yellow.

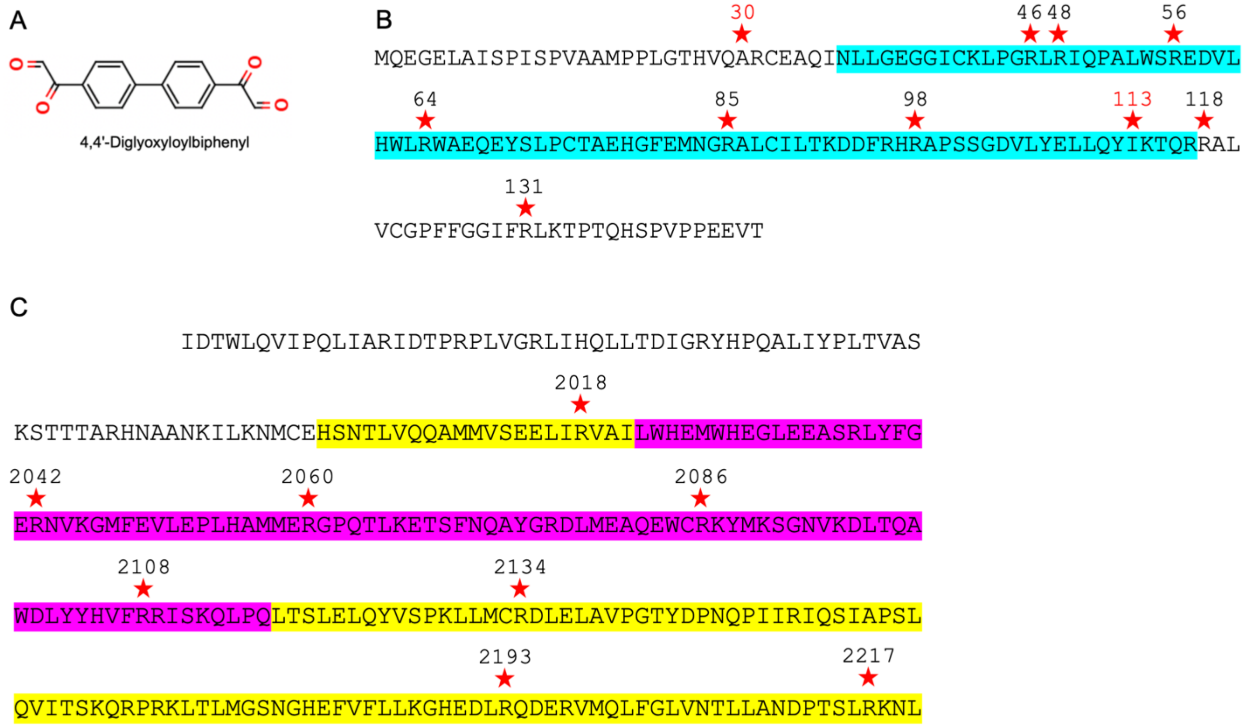

Supplemental Figure S4.

Chemical structure of the arginine cross-linker and cross-linked amino acids in the ETV7 PNT and mTOR FRB domains.

4A. Chemical structure of arginine crosslinker 4, 4'-diglyoxyloylbiphenyl.

4B. Position of cross-linked arginine amino acids in ETV7 marked by a star. Amino acid numbers are relative to the methionine at position 1, numbers in red indicate non-arginine crosslinks. Pointed domain sequences are highlighted in aqua.

4C. Position of cross-linked amino acids in the FRB fragment of mTOR marked by a star. Amino acid numbers are relative to the methionine at position 1. The N-terminal kinase lobe is highlighted in yellow, within these sequences the FRB domain is highlighted in fuchsia.

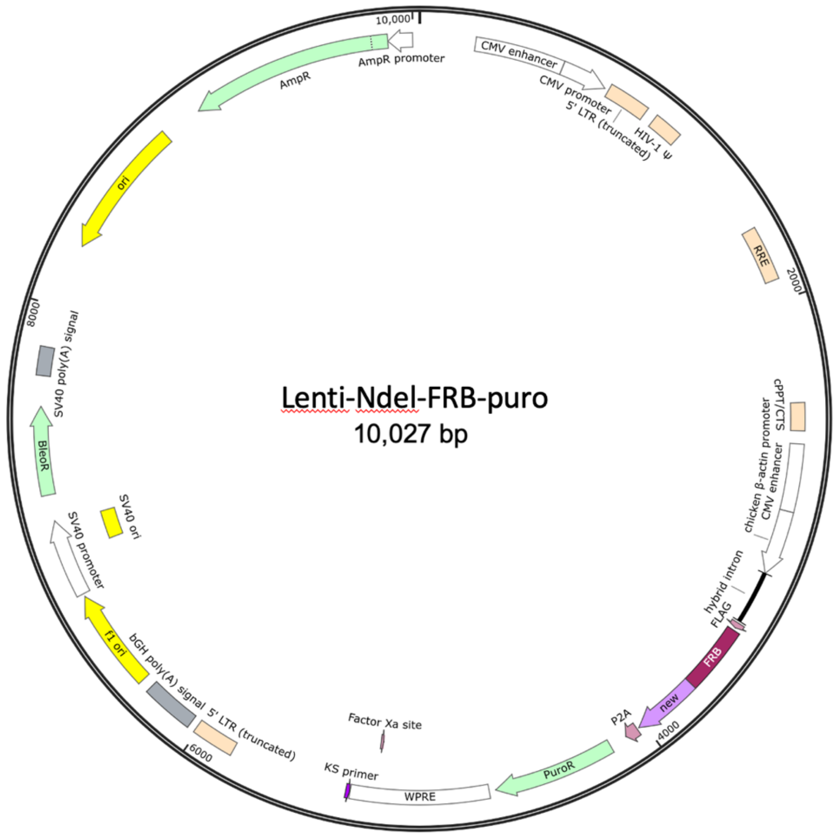

Supplemental Figure S5. Lenti-NdeI-FRB-puro.

Map of the lentiviral vector used for the expression of NdeI-FRB in Karpas-299 cells.

Supplemental Table S1. ΔΔG values for Alanine substitutions in the ETS domain of ETV7.

mutation	FoldX stability (kcal/mol)	ΔΔG (kcal/mol)
WT	-30.04	-
W227	-29.31	0.73
Y231	-28.88	1.16
L235	-29.29	0.75
Y239*	-27.69	2.35
Y242	-27.63	2.41
L264	-28.12	1.92
L289*	-29.29	0.75
I291	-28.26	1.78
L305	-29.06	0.98

*Alanine mutations that disrupt reciprocal co-IP

The stability of alanine mutants of the ETS domain of ETV7 as calculated by foldX with respect to the wild-type Alpha Fold model (Q9Y603), encompassing residues 223-305.
